# Safety, immune lot-to-lot consistency and non-inferiority of a fully liquid pentavalent DTwp-HepB-Hib vaccine in healthy Indian toddlers and infants

**DOI:** 10.1080/21645515.2015.1100779

**Published:** 2015-11-18

**Authors:** Dulari J. Gandhi, Sangappa M. Dhaded, Mandyam D. Ravi, Anand P. Dubey, Ritabrata Kundu, Sanjay K. Lalwani, Jugesh Chhatwal, Leni G. Mathew, Madhu Gupta, Shiv D. Sharma, Sandeep B. Bavdekar, Midde V. Jayanth, Suresh Ravinuthala, Arijit Sil, Mandeep S. Dhingra

**Affiliations:** a Department of Pediatrics, SBKS Medical College, Vadodara, India; b Department of Pediatrics, Jawaharlal Nehru Medical College, Belgaum, India; c Department of Pediatrics, JSS Medical College, Mysore, India; d Department of Pediatrics, Maulana Azad Medical College, Delhi, India; e Department of Pediatrics, Institute of Child Health, Kolkata, India; f Department of Pediatrics, Bharati Vidyapeeth Deemed University Medical College, Pune, India; g Department of Pediatrics, Christian Medical College, Ludhiana, India; h Department of Pediatrics, Christian Medical College, Vellore, India; i Department of Community Medicine, School of Public Health, Post Graduate Institute of Medical Education & Research, Chandigarh, India; j Department of Pediatrics, Sawai Man Singh Medical College, Jaipur, India; k Department of Pediatrics, Topiwala Nair Medical College, Mumbai, India; l Shantha Biotechnics Private Limited, Hyderabad, India

**Keywords:** DTwP-HepB-Hib, India, immunogenicity, lot-to-lot consistency, pentavalent vaccine, safety, whole-cell pertussis

## Abstract

Pentavalent combination vaccines are important tools to strengthen the immunization programs in numerous countries throughout the world. A large number of countries have recognized the value of combination vaccines and have introduced whole cell pentavalent vaccines into their immunization programs. A phase III, multi-center, randomized, single blinded study of a fully liquid pentavalent DTwP-HepB-Hib investigational vaccine (Shan5™) was conducted across India in 2 cohorts: 15 toddlers were evaluated for safety and immunogenicity following a single booster dose (Cohort 1) followed by 1085 infants (Cohort 2) evaluated for immunogenicity and safety following 3-dose primary immunization of the investigational vaccine or a locally licensed comparator vaccine (Pentavac SD). Immune consistency analysis among 3 lots of the investigational vaccine, and immune non-inferiority analysis of pooled (3 lots) data of investigational vaccine vs. comparator vaccine were carried out in cohort 2. The vaccines demonstrated comparable safety and immune responses in cohort 1. In cohort 2, equivalent immune consistency among 3 lots was observed for all antigens except whole cell pertussis antigens, where a marginal variation was observed which was linked to the low power of the test and concluded to not have any clinical significance. Immune non-inferiority against the comparator vaccine was demonstrated for all 5 antigens. Safety results were comparable between vaccine groups. This investigational, fully-liquid, whole-cell pertussis (wP) containing new pentavalent vaccine was found to be safe and immunologically non-inferior to the licensed comparator vaccine.

## Introduction

The use of combination vaccines to immunize against several diseases simultaneously is a recognized strategy for increasing vaccine coverage in childhood vaccination programs. By reducing the number of injections, compliance to vaccination is improved along with positive impact on administrative and logistic costs incurred while implementing vaccination programs.^1^ In the emerging economies of the world, cost and consistent availability of a combination vaccine are major hurdles for the introduction of such vaccines in the national immunization programs. An ideal combination vaccine should be safe, immunogenic, cost effective, easy to store and use, with antigenic components aligned with the recommended immunization schedule.^2^ With the aim to develop an ideal combination vaccine which can be included in the Indian national immunization programs, this fully liquid formulation of combined Diphtheria, Tetanus, whole-cell Pertussis, Hepatitis B and *Haemophilus influenzae* type b (DTwP-HepB-Hib) vaccine (Shan5™) has been developed. This phase III study was conducted in India to describe the safety and immunogenicity of a single dose of vaccine in toddlers followed by evaluation in infants of immune consistency among 3 lots of the investigational vaccine; immune non-inferiority of investigational vaccine (data pooled from 3 lots) as compared to a locally licensed DTwP-HepB-Hib pentavalent combination vaccine and describe the safety.

## Results

In cohort 1 (toddlers), 10 doses of investigational and 5 doses of comparator vaccine were administered as a single booster. In cohort 2 (infants), overall 2690 doses of investigational vaccine (930 first doses, 890 second doses and 870 third doses) and 447 doses of comparator vaccine (155 first doses, 148 second doses and 144 third doses) were administered.

### Lot-to lot consistency

Immune lot-to-lot consistency analysis revealed that for each valence, the observed inter-lot differences lie between 95% CI i.e. –δ to +δ (here δ = 10%) with the exception of anti-wP antibody levels for Lot A vs. Lot B and Lot B vs. Lot C pair, which were marginally out of specification. The detailed immune lot-to-lot consistency analysis results of cohort 2 are tabulated in Table 1.

**Table 1.** Lot-to-lot-consistency among investigational vaccine lots, non-inferiority for pooled investigational vaccine vs. comparator and post-Dose 3 GMT of pooled investigational vaccine vs. comparator as per per-protocol analysis set in cohort 2.

	Lot-to lot-consistency							
	Difference in seroresponse/seroprotection (%) and 95%[CI] (2- sided)			Non-inferiority [Seroprotection/Seroresponse rate; (95% CI)]			Post-dose 3 GMT	
Antibody	Lot A vs. Lot B	Lot A vs. Lot C	Lot B vs. Lot C	Pooled investigational vaccine (N = 822)	Comparator (N = 132)	Difference (%) and 95% CI (2 -sided)	Pooled investigational vaccine (N = 822)	Comparator (N = 132)
							GM (95% CI)	GM (95% CI)
Anti-wcP (NTU)	5.183 (−2.5; 12.8)	0.387 (−7.1; 7.9)	−4.796 (−12.3; 2.8)	70.1 (66.8; 73.2)	68.9 (60.3; 76.7)	1.146 (−6.802; 10.017)	17.5 (16.3; 18.6)	17.2 (14.7; 20.2)
Anti-Hib (mcg/mL)	−0.036 (−2.0; 1.9)	−0.758 (−2.7; 0.7)	−0.722 (−2.5; 0.7)	99.5 (98.8; 99.9)	100.0 (97.2; 100.0)	−0.488 (−1.249; 2.355)	8.87 (8.28; 9.51)	9.45 (7.77; 11.5)
Anti-HBs (mIU/mL)	−0.829 (−3.4; 1.5)	1.681 (−1.2; 4.7)	2.510 (−0.1; 5.4)	97.8 (96.5; 98.7)	100.0 (97.2; 100.0)	−2.209 (−3.464; 0.752)	836 (750; 932)	1908 (1477; 2466)
Anti-D (IU/mL)	0.000 (−1.4; 1.3)	0.000 (−1.4; 1.3)	0.000 (−1.3; 1.3)	100.0 (99.6; 100.0)	100.0 (97.2; 100.0)	0.000 (−0.467; 2.828)	1.61 (1.52; 1.69)	1.46 (1.26; 1.69)
Anti-T (IU/mL)	0.000 (−1.4; 1.3)	0.000 (−1.4; 1.3)	0.000 (−1.3; 1.3)	100.0 (99.6; 100.0)	100.0 (97.2; 100.0)	0.000 (−0.467; 2.828)	1.85 (1.74; 1.98)	2.08 (1.78; 2.43)

N= Number of subjects analyzed according to PP analysis Set

### Immunogenicity analysis

In cohort 1, the seroprotection rates for toddlers pertaining to Diphtheria, Pertussis and Hib were 100% and seroresponse rate for whole cell pertussis was 80% in both study groups. Hepatitis B seroprotection rates were 90% and 100% in investigational and comparator group respectively. The detailed immunogenicity results of cohort 1 are tabulated in Table 2.

**Table 2.** Seroprotection/seroresponse rate & Geometric Mean Concentrations of cohort 1.

		Investigational Vaccine (N = 10)				Comparator vaccine (N=5)			
Antibody	Criteria	n/M	%	95% CI	Post Dose GMC	n/M	%	95% CI	Post Dose GMC
Anti-wP	> 11 NTU	8/10	80.0	(44.4; 97.5)	21.8	4/5	80.0	(28.4; 99.5)	20.5
Anti-Hib	≥ 0.15 mcg/mL	10/10	100.0	(69.2; 100.0)	7.73	5/5	100.0	(47.8; 100.0)	5.72
Anti-HBs	≥ 10 mIU/mL	9/10	90.0	(55.5; 99.7)	1293	5/5	100.0	(47.8; 100.0)	2018
Anti-D	≥ 0.01 IU/mL	10/10	100.0	(69.2; 100.0)	2.12	5/5	100.0	(47.8; 100.0)	2.40
Anti-T	≥ 0.01 IU/mL	10/10	100.0	(69.2; 100.0)	17.1	5/5	100.0	(47.8; 100.0)	26.8

M: number of subjects with available data for the relevant endpoint; n: number of subjects experiencing endpoint listed in the criteria column; %: percentages and 95% CI are calculated according to the subjects available for the endpoint.

In cohort 2, the seroprotection rates for infants pertaining to Diphtheria and Tetanus were 100% in both study groups. Seroprotection rates of investigational and comparator group for Hepatitis B and Hib were 97.8% vs. 100% and 99.5% vs. 100% respectively. Seroresponse rate of investigational and comparator group for whole cell pertussis was 70.1% vs. 68.9%. The detailed immunogenicity results of cohort 2 are tabulated in Table 1. Following three doses of primary vaccination (cohort 2), 96.7% (95% CI 95.2; 97.8) of subjects in the investigational vaccine groups (pooled lots) and 95.5% (95% CI 90.4; 98.3) of subjects in the comparator vaccine group had anti-PRP antibody titers more than 1.0 mcg per mL (considered a marker of long term protection). Similarly 91.2 % (95% CI 89.0; 93.0) of subjects in the investigational vaccine groups (pooled lots) and 95.4% (95% CI 90.3; 98.3) of subjects in the comparator group had post vaccination anti-HBS antibody titres more than 100 mIU per mL (considered a marker of long-term protection). The percentage of subjects exhibiting a post vaccination titer more than 1.0 IU per mL for Diphtheria and Tetanus antibodies in the investigational vaccine groups (pooled lots) were 80.5% (95% CI 77.6; 83.1) and 76.3% (95% CI 73.2; 79.2) respectively. The corresponding values for the comparator group were 76.5% (95% CI 68.4; 83.5) and 78.0% (95% CI 70.0; 84.8) for Diphtheria and Tetanus respectively.

**Figure 1.**
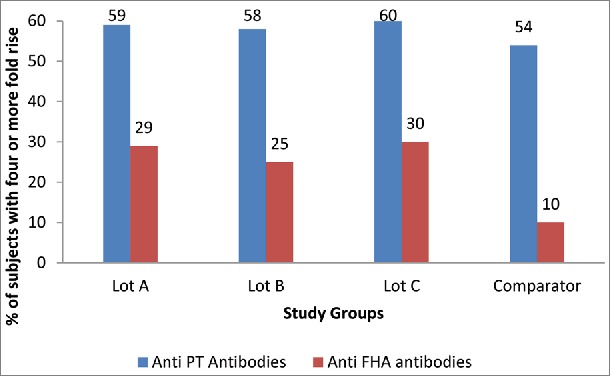
Percentage of subjects in cohort 2 demonstrating a 4 Fold or more rise of anti-PT and anti-FHA antibody titers (Lot A, B and C refer to investigational vaccine).

Four-fold or more rise for anti-pertussis toxin (anti-PT) and anti-filamentous hemagglutinin (anti-FHA) titers (vs baseline) was observed in 59% vs. 54% and 28% vs. 10% subjects in investigational (pooled lots) and comparator group respectively (Fig. 1).

### Geometric mean concentrations

**Figure 2.**
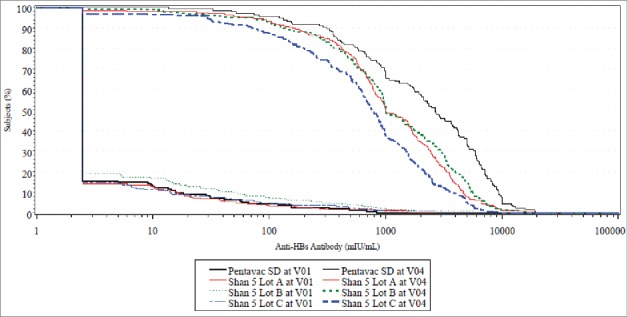
RCDC (Reverse Cumulative Distribution Curves) for all investigational vs. comparator Pre-Primary and Post-Dose 3 in cohort 2 for Anti HBS antibody response.

In cohort 2, Geometric Mean Concentrations of 5 antigens following investigational (pooled lots) and comparator vaccine administrations respectively were as follows; Diphtheria (1.61 IU/mL and 1.46 IU/mL), Tetanus (1.85 IU/mL and 2.08 IU/mL), Pertussis (17.5 NTU and 17.2 NTU), Hib (8.87 mcg/mL and 9.45 mcg/mL) and Hepatitis B (836 mIU/mL and 1908 mIU/mL). The detailed Geometric Mean Concentrations for cohort 2 are tabulated in Table 1 and Figure 2, and for cohort 1 in Table 2.

### Safety analysis

In cohort 1, solicited injection site reactions were observed in 60% (6 subjects) and 80% (4 subjects); systemic reactions were observed in 50% (5 subjects) and 60% (3 subjects) whereas unsolicited reactions were observed in 40% (4 subjects) and 20% (1 subject) in investigational and comparator groups respectively. No Serious Adverse Events were observed.

In cohort 2, tenderness at the site of injection was the most frequently reported solicited injection site reaction across all doses for the 2 study groups followed by swelling and erythema. Crying excessively was the most frequently reported solicited systemic reaction across all doses for the 2 study groups followed by irritability and erythema. Dose-wise incidence of all solicited adverse events according to severity (any grade and grade 3) in cohort 2 is summarized in Table 3. The most frequently reported unsolicited events were of “infection and infestation category” (investigational: 7.2% and comparator: 8.4%) followed by “respiratory, thoracic and mediastinal disorder” and gastrointestinal disorder. Total of Six (6) serious adverse events, including 2 fatalities were reported from the study, all the SAEs were observed in Shan5 group. None of these events were considered as related to vaccine administration. Out of these 6 SAEs, 2 were reported during the 6 months follow up period post third dose (Table 4). All the SAEs were reviewed by the Data Safety Monitoring Board and reported to the National Regulatory Authority of India, Ethics Committee at the site of occurrence and the ethics committees at the other sites in the study as per GCP and Indian Regulatory requirements. The two fatal cases were also reviewed at the level of an independent expert committee constituted by the Indian regulators and confirmed as not related to vaccination.

**Table 3.** Incidence of solicited adverse reactions in cohort 2 as observed over 28 d of follow up period after each dose.

		Dose 1		Dose 2		Dose 3	
Solicited Adverse Reactions	Severity	Pooled investigational vaccine N = 930 M = 901 n*(%)	Comparator N = 155 M = 151 n*(%)	Pooled investigational vaccine N = 890 M = 873 n*(%)	Comparator N = 148 M = 144 n*(%)	Pooled investigational vaccine N = 870 M = 846 n*(%)	Comparator N = 144 M = 137 n*(%)
**Injection Site Reactions**							
**Tenderness**	Any grade	564 (62.6)	91 (60.3)	538 (61.6)	82 (56.9)	453 (53.5)	78 (56.9)
	Grade 3	121 (13.4)	18 (11.9)	116 (13.3)	24 (16.7)	58 (6.9)	16 (11.7)
**Erythema**	Any grade	209 (23.2)	29 (19.3)	225 (25.8)	28 (19.6)	194 (22.9)	33 (24.3)
	Grade 3	3 (0.3)	0 (0.0)	1 (0.1)	0 (0.0)	2 (0.2)	0 (0.0)
**Swelling**	Any grade	346 (38.4)	54 (36.2)	321 (36.8)	46 (32.2)	253 (29.9)	43 (31.6)
	Grade 3	4 (0.4)	2 (1.3)	1 (0.1)	0 (0.0)	0 (0.0)	1 (0.7)
**Systemic Reactions**							
**Fever**	Any grade	217 (24.1)	42 (28.0)	275 (31.5)	61 (42.4)	213 (25.2)	43 (31.4)
	Grade 3	4 (0.4)	0 (0.0)	7 (0.8)	1 (0.7)	4 (0.5)	0 (0.0)
**Vomiting**	Any grade	112 (12.4)	15 10.0)	102 (11.7)	18 (12.5)	57 (6.7)	6 (4.4)
	Grade 3	9 (1.0)	0 (0.0)	3 0.3)	0 (0.0)	2 (0.2)	0 (0.0)
**Crying abnormal**	Any grade	421 (46.8)	71 (47.3)	397 (45.5)	68 (47.2)	298 (35.2)	57 (41.6)
	Grade 3	42 (4.7)	5 (3.3)	37 (4.2)	9 (6.3)	16 (1.9)	9 (6.6)
**Drowsiness**	Any grade	190 (21.1)	38 (25.3)	180 (20.6)	30 (20.8)	114 (13.5)	25 (18.2)
	Grade 3	24 (2.7)	2 (1.3)	14 (1.6)	2 (1.4)	7 (0.8)	1 (0.7)
**Appetite lost**	Any grade	205 (22.8)	31 (20.7)	205 (23.5)	36 (25.0)	132 (15.6)	28 (20.4)
	Grade 3	14 (1.6)	6 (4.0)	14 (1.6)	4 (2.8)	15 (1.8)	4 (2.9)
**Irritability**	Any grade	343 (38.1)	46 (30.7)	331 (37.9)	61 (42.4)	243 (28.7)	50 (36.5)
	Grade 3	28 (3.1)	2 (1.3)	24 (2.7)	7 (4.9)	9 (1.1)	1 (0.7)

*n = number subjects with incidence of events; M = number of subjects for which the safety data is available; N = number of doses administered

**Table 4.** Details of SAEs observed in cohort 2.

SAE Number	Vaccine administered	Immunization number	Time after immunization	Complaint/Diagnosis	Outcome	Relationship
1	Shan 5 (Lot B)	Dose 2	25 d post dose 2	Pneumonia	Death	Not Related
2	Shan 5 (Lot A)	Dose 1	29 d post dose 1	Metabolic Acidosis	Death	Not Related
3	Shan 5 (Lot C)	Dose 1	6 d post dose 1	Aspiration/Laryngospasm /Convulsions	Recovered	Not Related
4	Shan 5 (Lot B)	Dose 1	24 d post dose 1	Benign Intracranial Hypertension	Recovered	Not Related
5	Shan 5 (Lot C)	Dose 3	144 d (or 5 months) post dose 3	Arthropod Sting	Recovered	Not Related
6	Shan 5 (Lot B)	Dose 3	154 d (or 5.5 months) post dose 3	Lower Respiratory Tract Infection	Recovered	Not Related

**Table 5.** Kit/Lab developed Assay and the cut-off values of seroprotection/seroresponse for different antibodies.

Antibody	Kit/Lab developed assay	Cut-off values of seroprotection /seroresponse
Anti-wcP	NovaLisa™ Bordetella pertussis IgG – ELISA, Novatec Immundiagnostica GMBH, Germany	>11 NTU*
Anti-Hib	VACCZYME, anti Haemophilus influenzae type b enzyme immunoassay kit, Binding Site Limited, Birmingham UK	≥0.15 μg/mL
Anti-HBs	VITROS Anti-HBs Reagent Pack, Ortho Clinical Diagnostics, Johnson & Johnson, USA	≥10 mIU/mL
Anti-D	NovaLisa™ *Corynebacterium diphtheriae* toxin IgG-ELISA, Novatec Immundiagnostica GMBH, Germany	≥0.01 IU/mL
Anti-T	Tetanus IgG ELISA, IBL International GmbH, Germany	≥0.01 IU/mL
Anti-PT	Assay developed by Focus lab	> 45 IU/mL
Anti-FHA	Assay developed by Focus lab	> 90 IU/mL

*A correlate of protection has yet to be established for pertussis11, therefore seroconversion (for primary objective) was defined as a post vaccination titer more than or equal to the pre-vaccination titer in initially seropositive subjects (>11 NTU) and in case of initial seronegative subjects (≤ 11 NTU), the response was considered according to assay cut off (>11 NTU).

## Discussion

The present study evaluated safety and immunogenicity profiles between 2 study groups in cohort 1 (toddlers) and safety, immune consistency among 3 lots of the investigational vaccine and immune non-inferiority of investigational vaccine against comparator vaccine in cohort 2 (infants). The seroprotection and seroresponse rates observed in cohort 2 were within the expected ranges based on data published from India where other DTwP-HepB-Hib pentavalent and tetravalent vaccines have been evaluated in various clinical studies.^3–7^

**Figure 3.**
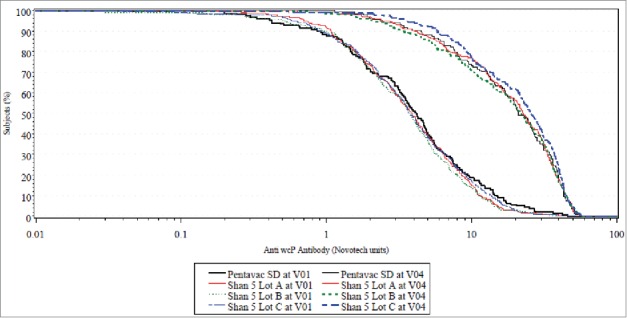
RCDC (Reverse Cumulative Distribution Curves) for all investigational vs. comparator Pre-Primary and Post-Dose 3 in cohort 2 for whole cell pertussis antibody response.

In cohort 2, the primary objective of immune lot-to-lot consistency among 3 lots was met for 4 antigens viz. Hib (PRP), Hepatitis B, Diphtheria and Tetanus. For whole cell pertussis (wP), the equivalence tests were marginally outside of defined expected target specification (i.e., δ = ± 10%) for 2 (Lot A vs. Lot B and Lot B vs. Lot C) out of 3 tests. This marginal miss can be attributed to the low ‘a posteriori’ power of the test (21.2%) as opposed to the ‘a priori’ target power (82%). The target assumed for seroresponse rate of wP was 89% based on our previous experience with the same type of vaccine but using a different ELISA kit.^3^ The observed seroresponse for wP for all 3 lots of investigational vaccine [Lot A (72%), Lot B (66.8%), Lot C (71.6%)] were lower than the target. This likely may have resulted due to the change in ELISA kit. This observed seroresponse resulted in a lower power of the test which makes it difficult to definitely conclude on lot to lot consistency for wP given the marginal miss [95% CI for Lot A vs. Lot B is (−2.59; 12.85) and for Lot B vs. Lot C is (−12.39; 2.88)]. However, the marginal miss to achieve the pre-specified statistical margins for lot to lot consistency has limited clinical relevance. The Reverse Cumulative Distribution Curves for antibody response of 3 lots of investigational vaccine display the entire distribution of wP titers observed in the study participants (Fig. 3). The curves are very similar across the entire range suggesting consistency and minimal variability. Additionally, when a formal post hoc statistical comparison was performed (log rank test), the findings were non-significant (p = 0.3017) demonstrating minimal heterogeneity of the distribution. Additionally the observed seroresponse rate for the comparator vaccine [68.9% (95% CI 60.3; 76.7)] was similar and statistically non-inferior to the seroresponse observed with the investigational vaccine.

The anti-PT antibody response (Lot A: 36.7%, Lot B: 37.7% and Lot C: 41.2%) and anti-FHA antibody response (Lot A: 3.8%, Lot B: 2.5% and Lot C: 3.1%) were also similar across all the 3 lots of investigational vaccine. This further strengthens the rationale that the marginal miss in lot to lot consistency was predominately influenced by the low power of the test and thus, is unlikely to be a true observation.

In cohort 2, the co-primary objective of immune non-inferiority was established for all 5 antigens where all the antigens met the pre-specified criteria for immune non-inferiority i.e., for each valence, the lower limit of the 95% CI of the observed difference was greater than –δ (−10%) and thus pooled lots was considered to be non-inferior to comparator vaccine.

Comparable safety profile was observed between both groups of Cohort 1. In cohort 2; Immediate, solicited injection site and systemic reactions were similar in subjects receiving investigational and comparator vaccine. None of the unsolicited adverse reports reported in the study were related to any of the vaccine and resolved with or without any medications. All the SAEs (including the fatalities) were confirmed as un-related to either of the study product by the investigators and the Independent Data Safety Monitoring Board.

## Patients and methods

### Study design

A Multi-center (11 study sites), randomized, single blinded study was conducted initially in 15 toddlers who were followed up for safety and tolerability for 28 d following single booster dose (Cohort 1) followed by evaluation of 1085 infants followed up for immunogenicity for 28 d and safety for 6 months following 3 doses of the vaccine administered at 6–8, 10–12 and 14–16 weeks of age (Cohort 2). The study was designed as a 2-arm trial in cohort 1 (investigational: comparator - 2:1 randomization) and a 4-arm trial of 3 lots of investigational vaccine (Lot A, Lot B and Lot C) and one arm receiving comparator vaccine in Cohort 2 (2:2:2:1 randomization). Protocols and the other relevant study documents were approved by the Drugs Controller General of India (DCGI) and respective institutional ethics committees prior to the start of the study. Study was conducted according to the guidelines laid in Declaration of Helsinki and Good Clinical Practice as per ICH, Indian GCP and applicable regulatory guidelines. Subjects were recruited following a written informed consent provided by parents or legally acceptable representative (LAR). The study was registered in the Clinical Trial Register of India (Registration number: CTRI/2012/08/002872).

### Study participants

Healthy toddlers aged 15–18 months were enrolled in cohort 1 based upon their primary immunization profile. Healthy infants aged 6–8 weeks were enrolled in Cohort 2 based upon the gestational age (37 completed weeks at birth), mother's medical history and weight at birth (≥2.5 kg) unless possibly allergic to any component of the vaccine, or immunized previously with similar vaccines except BCG, birth dose of Hepatitis B and/or OPV. Infants who had been administered a birth dose of the hepatitis B vaccine, as per the study center protocol, were not excluded from the study but a record of this was made in the CRFs.

**Figure 4.**
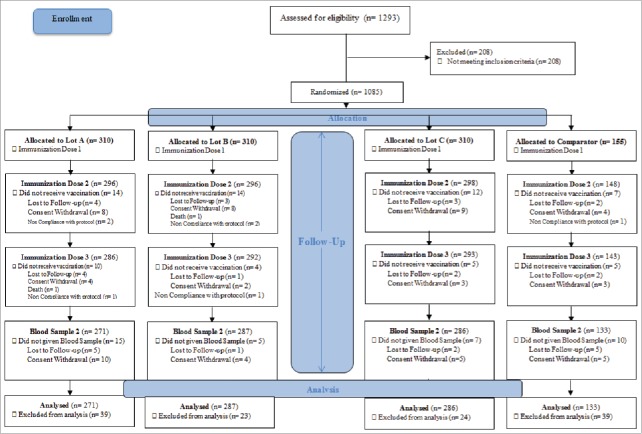
Cohort 2 Subject Disposition Flow Chart.

All the 15 subjects enrolled in cohort 1, completed the study. In Cohort 2, a total of 954 subjects completed the study as per protocol out of total 1085 subjects recruited in the study. Subject disposition of Cohort 2 is summarized in Figure 4. The mean age of vaccinees at recruitment was 16.5 months and 6.8 weeks for cohort 1 and cohort 2 respectively. The study groups were comparable for other baseline characteristics (male to female ratio, birth weight, height, head circumference and body mass index) at enrollment.

### Study vaccines

One lot of investigational (PLK003A11) and comparator (137P1017E) vaccines were used in Cohort 1 whereas 3 lots of investigational [Lot A: PLK002A11 (manufactured Nov 2011), Lot B: PLK003A11 (manufactured Nov 2011) and Lot C: PLK004A11(manufactured Dec 2011)] and one batch of comparator (137P1017A) vaccines were used in Cohort 2. Each dose (0.5 mL) of investigational vaccine contains diphtheria toxoid (≥30 IU), tetanus toxoid (≥60 IU), whole cell *Bordetella pertussis* (≥4 IU), HBV surface antigen (10 mcg), Hib polysaccharide conjugated with tetanus toxoid (10 mcg), adsorbed on Aluminum Phosphate (0.625 mg) as adjuvant, Thiomersal as preservative (0.050 mg) along with sodium chloride (4.5 mg) and the volume was made 0.5 mL with water for injection. A single dose (0.5 mL) of comparator vaccine contains diphtheria toxoid (≥20 Lf to ≤30 Lf), tetanus toxoid (≥2.5 Lf to ≤10 Lf), whole cell *Bordetella pertussis* (≥4 IU), HBV surface antigen (≥ 10 mcg), conjugated Hib polysaccharide (10 mcg), adsorbed on Aluminum Phosphate (≤1.25 mg) as adjuvant and Thiomersal 0.005%.

### Objectives

The study was conducted in 2 cohorts. A smaller initial group of 15 toddlers followed up for 28 d post single dose of vaccine administered as a booster (cohort 1) followed by a larger group of 1085 infants administered 3 doses of vaccine as a primary series and followed up for 6 months (cohort 2). In cohort 1, the primary objective was to assess the safety of investigational vaccine as compared to comparator vaccine up to a follow-up period of 28 d post the booster dose. The secondary objective in cohort 1 was to describe the immunogenicity of all 5 antigens as evaluated 28 d post booster dose. In Cohort 2, the primary objective was to demonstrate the equivalence of immunogenicity among 3 lots of investigational vaccine to all the 5 antigens, one month after a 3-dose primary series in terms of seroprotection rates for D, T, Hep B, and Hib antibodies; seroresponse rates for wP antibodies. A co-primary objective was to demonstrate the non-inferiority of pooled investigational vaccine in comparison to comparator vaccine in terms of seroprotection/seroresponse rates for all antigens, one month after a 3-dose primary series. Descriptive evaluation of safety, up to 28 d after each vaccination and 6 months post-Dose 3, was a secondary objective for cohort 2.

### Study procedures

The toddlers in cohort 1 were vaccinated with a single booster dose of either investigational or comparator vaccine whereas in cohort 2, infants were vaccinated with 3 doses of either the investigational vaccine (from one of the 3 lots) or comparator vaccine based upon the randomization list. Respective vaccines were injected intramuscularly in the toddler's/infant's upper thigh in a dose of 0.5 mL each. Alternate thighs were used for vaccination of each subsequent dose in infants. The vaccinated toddlers (Cohort 1) were observed for 30 minutes post-vaccination for any immediate adverse events and followed up for safety and immunogenicity for 28 d for solicited and unsolicited systemic and local adverse events. Similarly infants (Cohort 2) were observed for 30 minutes post-vaccination for any immediate adverse events and followed up for immunogenicity for 28 d and safety for 6 months following 3 doses of the vaccine. Parents or guardians recorded pre-specified local and systemic reactions, and unsolicited adverse events on dairy cards for 7 d and 28 d respectively following vaccination. Axillary temperature was measured daily using a standard electronic thermometer provided by the sponsor. Serious adverse events were documented from enrollment to last follow-up visit. All adverse events were reported and recorded as per Brighton case definitions issued by Brighton Collaboration.^13^ Following each dose of the vaccine, the infants with body temperature ≥40.4°C, persistent screaming or crying for 3 hours within 48 hours of vaccination, seizures, encephalopathy or hypersensitivity reaction were planned to be excluded from receiving subsequent doses of the vaccines.

Two (2) blood samples of approximately 5 mL each were collected, one just before the first dose of vaccine and another 28 d (with a window of 7 days) after the single booster dose or after the third dose of vaccine in cohort 1 and cohort 2 respectively. The trial sera were labeled with a unique identifier number at the study sites so that all the serological assays could be performed in a blinded manner. Antibodies were estimated for all antigens by Enzyme Linked Immunosorbent Assay (ELISA) except for anti-PT and anti-FHA, where the method was Luminex-based Multi Analyte Immuno Detection (MAID) Assay. All the assays were setup and validated at the laboratory prior to running the clinical samples and the operators were blinded to the vaccine received by the subjects. The Kit/Lab developed assay used and the cut-off values for seroprotection/seroresponse for different antibodies are tabulated in Table 5.^8-12^

### Statistical analysis

All statistical analysis was carried out with the SAS® software, version 8.2 or above (SAS Institute, Cary, NC, USA). No statistical hypotheses were tested for cohort 1 whereas different hypotheses were tested toward lot-to-lot consistency and non-inferiority for cohort 2.

Subjects were randomized with a block size of 3 (2:1 with 2 for investigational and 1 for comparator vaccine) in cohort 1 and with a block size of 14 (2:2:2:1 with 2 for each lots of investigational and 1 for comparator vaccine) in Cohort 2 using Proc Plan with SAS v9.2. In order to achieve an overall Global power of 82% with an assumption that only 90% of subjects will be evaluable on the per protocol set, a total of 1085 subjects were enrolled in Cohort 2.

For **lot-to-lot consistency**, a 3 paired equivalence testing approach was used to test seroprotection/seroresponse rate 4 weeks after the third dose of vaccination for each antigens with a pre-defined equivalence limit (δ) of 10%. The statistical methodology was based on the use of the 2-sided 95% confidence interval (CI) of the differences of the seroprotection/seroresponse rates between the pairs of lots for all antigens.

**Non-inferiority** of investigational vaccine (3 pooled lots) in terms of seroprotection/seroresponse rates were demonstrated if the overall null hypothesis is rejected, that is, individual null hypotheses for all antigens have to be rejected. The relevant limit for non-inferiority was planned as −10% for D, T, wP, Hep B, and Hib antigens. The statistical methodology was based on the lower bound of the 2-sided 95% CI of the difference of the seroprotection/seroresponse rates.

**For safety analysis**, a descriptive analysis of the reported solicited and unsolicited adverse events, as reported in diary cards or as noted by the study team, after each dose between 2 groups was undertaken.

Immunogenicity analysis was based on the ‘Per Protocol’ population (eligible immunized subjects who completed the study and provided pre and post vaccination blood samples) and the analysis was performed according to the randomized vaccine group. Safety analysis was based on ‘Safety Analysis Set (SafAS)’ population [All subjects who were given at least one dose of the investigational or comparator vaccine and for whom safety data/information are recorded (even if no symptom occurred)] and the analysis was performed according to the injected vaccine group.

## Conclusion

The study demonstrated the immune lot-to-lot consistency among investigational vaccine lots; immune non-inferiority of investigational vaccine against the locally licensed pentavalent vaccine in infants and a comparable immunogenicity profile in toddlers. It also demonstrated a comparable safety profile in infants as well as toddlers. Shan5™ was developed by Shantha Biotechnics Private Limited (A Sanofi Company) and benefitted from Sanofi Pasteur's more than 50 y of experience with whole-cell pertussis and combination vaccines. The vaccine meets all the requirements of an ideal combination vaccine i.e., consistent, safe, immunogenic, affordable and fully liquid to be ready for use. The vaccine received licensure and approval for marketing in India from the Drugs Controller General of India and received WHO-Pre Qualification status in April 2014 based on the results of this study.
